# Supplementary material for: Changing to a Low-Polyphenol Diet Alters Vascular Biomarkers in Healthy Men after Only Two Weeks
Source: Nutrients. 2018 Nov 14;10(11):1766. doi: 10.3390/nu10111766 (PMC6267476; doi:10.3390/nu10111766)
Supplement: Supplementary file 1 [file nutrients-10-01766-s001.pdf]

Table S1. Recommendations to follow a low antioxidant diet (LAD).

Restricted	Moderated	Allowed
**FOOD**		
Whole cereals and corn	Vegetables: garlic, onion, turnip, eggplant (with peel), artichoke, iceberg lettuce	Refined cereals (white rice, pasta…)
Cured cheese	Soy and derivatives (tofu…)	Meat
Yolk	Broad beans	Fish
Green leafy vegetables (spinach, Swiss chard, watercress…)	Fruits: pineapple, avocado, lemon, kiwi, banana	Skimmed dairy products (milk, yogurt…)
Vegetables: carrot, tomato broccoli, cabbages, pepper, leek, pumpkin, asparagus, green beans, beet, sweet potato	Olive oil	Egg white
Fruits: orange, tangerine, grapefruit, red fruits (strawberries, blueberries, blackberries, raspberries…), grape, pomegranate, plum, papaya, cantaloupe, persimmon, peach		Vegetables: eggplant (without peel), celery, zucchini, cauliflower, radish, potato, cucumber
Olives		Mushrooms
Green peas		Fruits: apple (without peel), pear (without peel), pineapple in syrup, figs
Nuts (almonds, walnuts…)		Legumes: white beans, chickpeas, lentils
Aromatic herbs (parsley, coriander, oregano…)		
Spices (turmeric, paprika…)		
Mustard		
Algae		
Cocoa		
**BEVERAGES**		
Infusions and teas	Coffee (maxim 1 coffee/day)	Water
Beer	Spirits	Carbonated water
Wine, cava or cider		Soda
Cocoa beverage		Chicken or fish broth (without vegetables)
Fruit juices		
Vegetables beverage (soymilk, almonds milk…)		
